# Supplementary figures and images for: Metabolite import by SLC33A1 is required for ATF6 activation during endoplasmic reticulum stress
Source: Life Sci Alliance. 2026 Apr 17;9(6):e202603679. doi: 10.26508/lsa.202603679 (PMC13090131; doi:10.26508/lsa.202603679)

Fig S2C

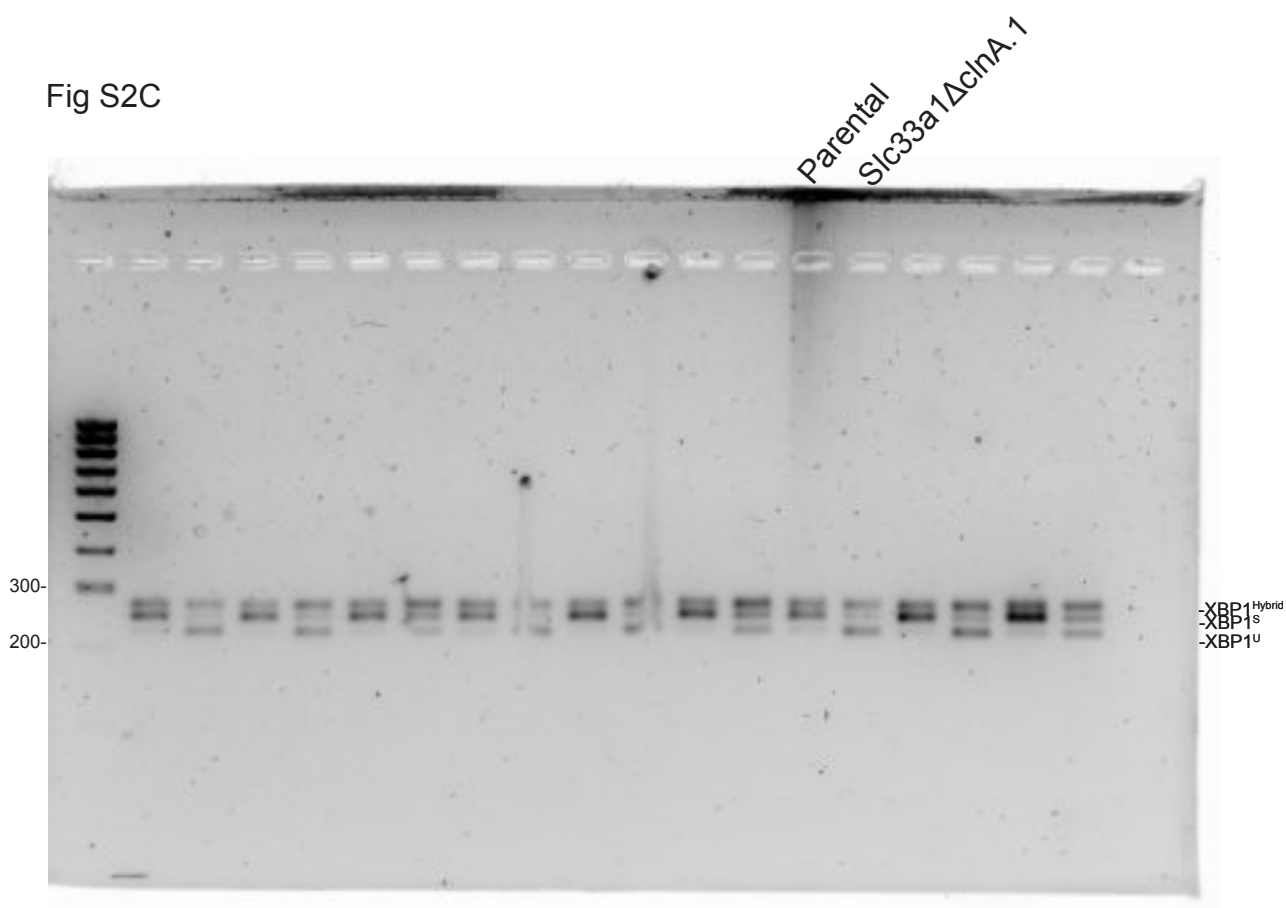

Supplement: Supplementary file 1 [file LSA-2026-03679_SdataFS2.pdf]

Fig 2B

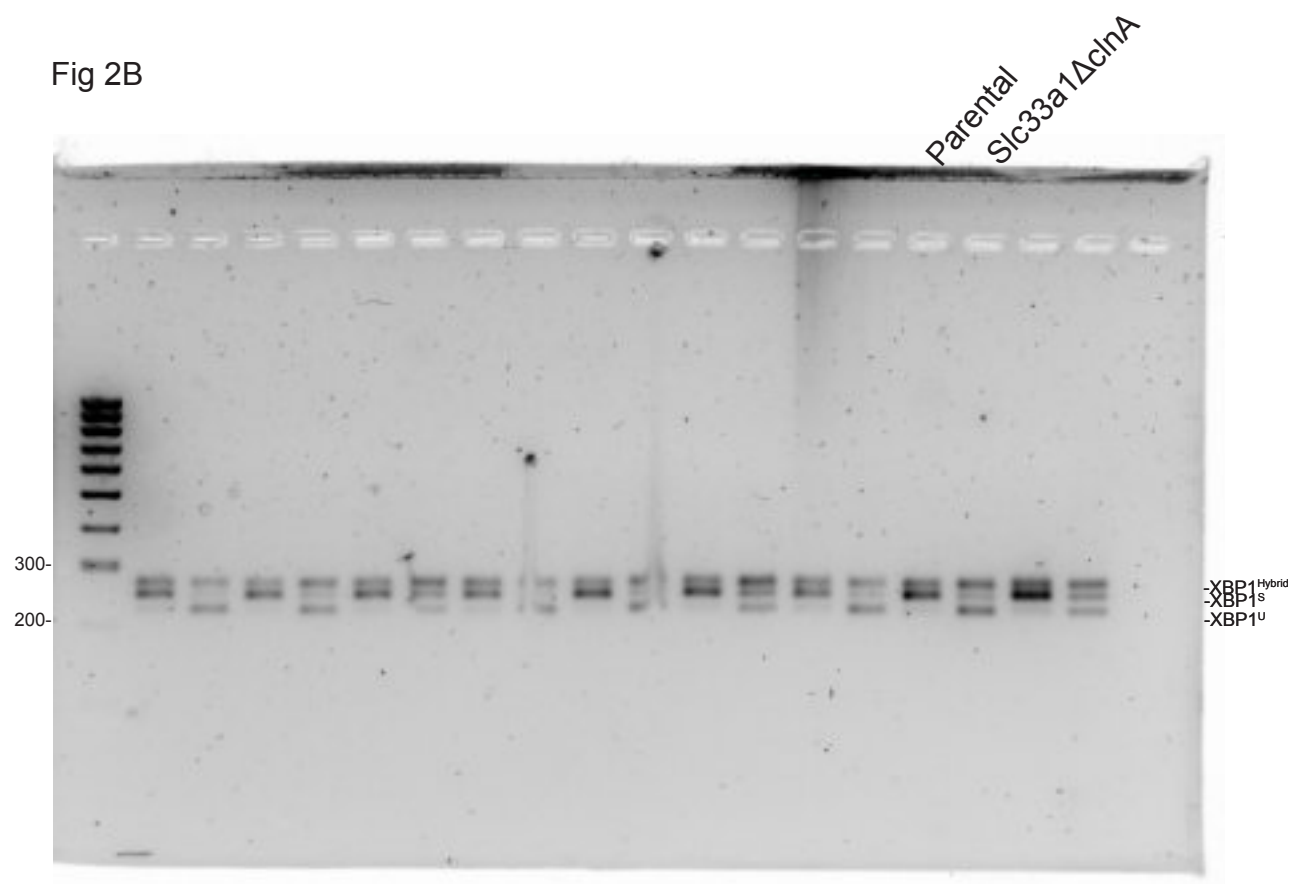

Supplement: Supplementary file 2 [file LSA-2026-03679_SdataF2.pdf]

Fig 3A

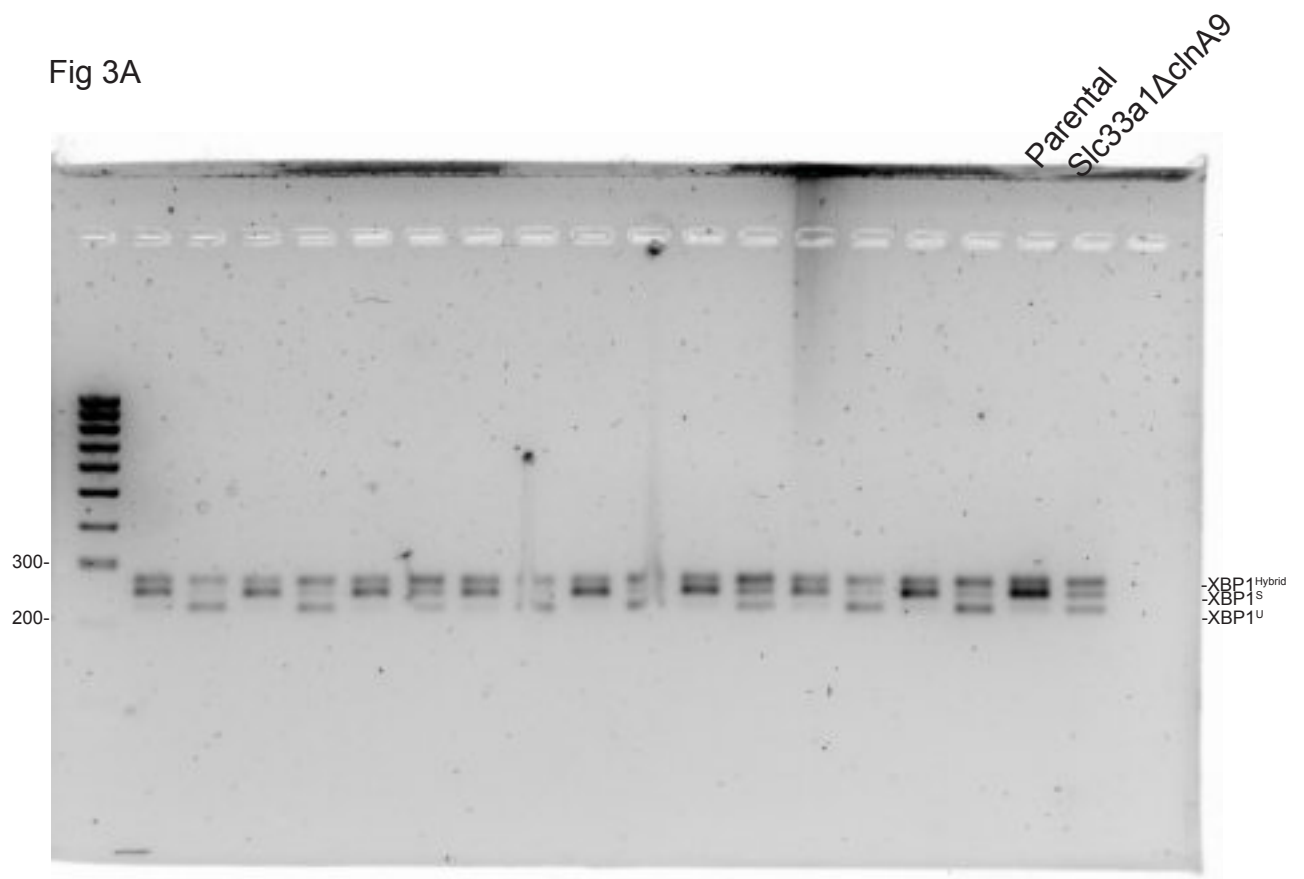

Fig 3B

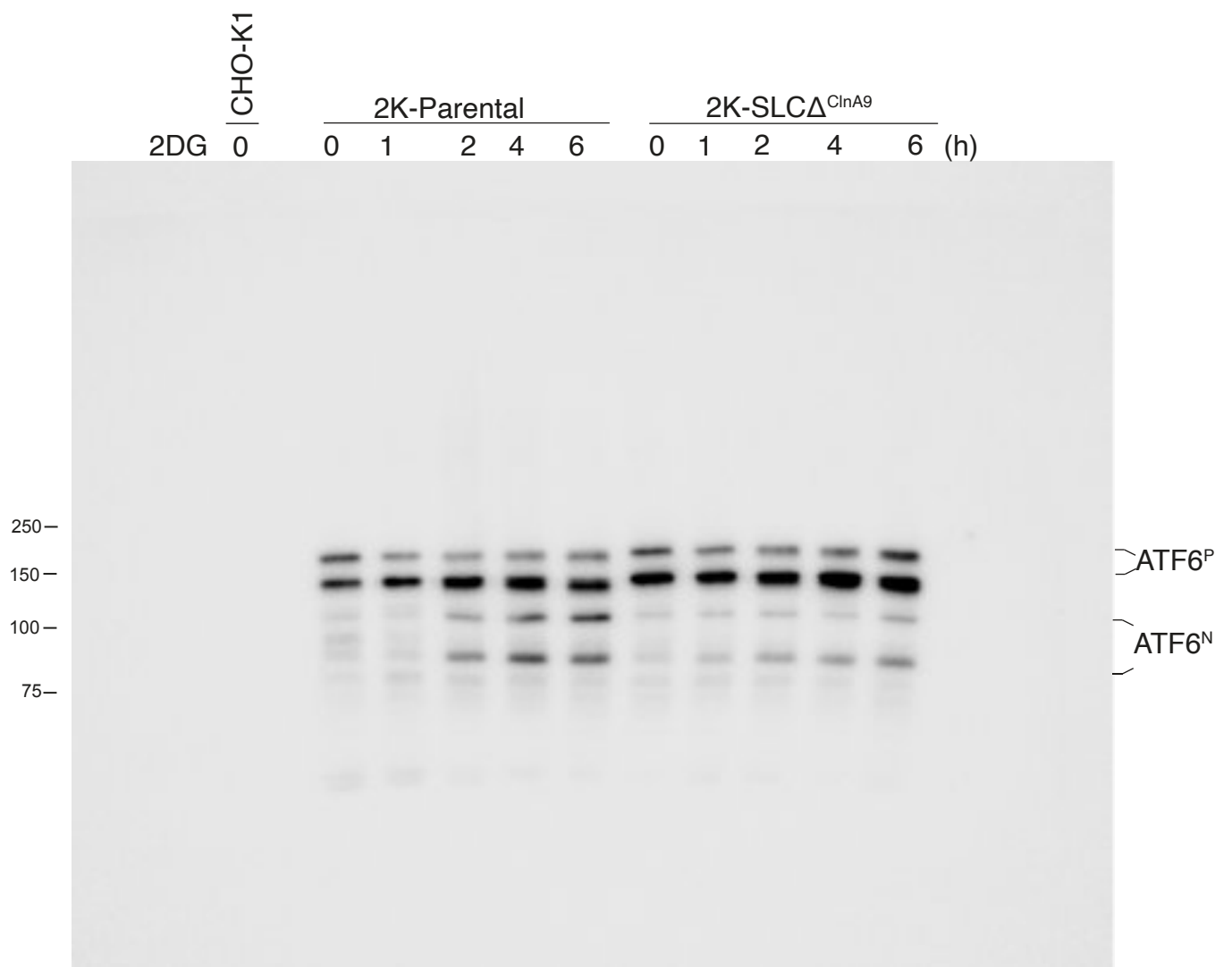

Supplement: Supplementary file 3 [file LSA-2026-03679_SdataF3.pdf]

Fig5B

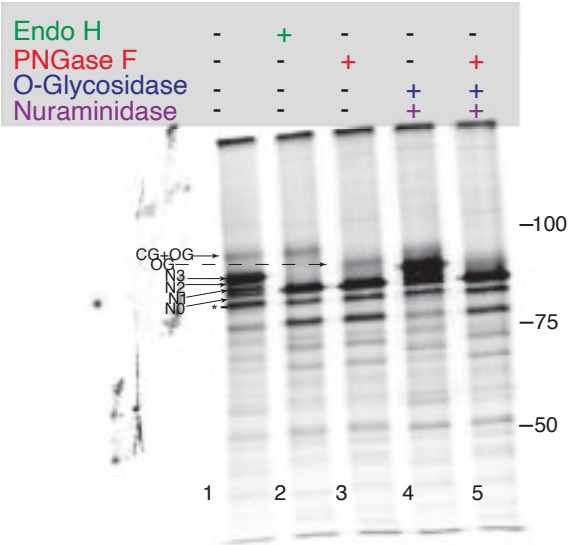

Fig5C

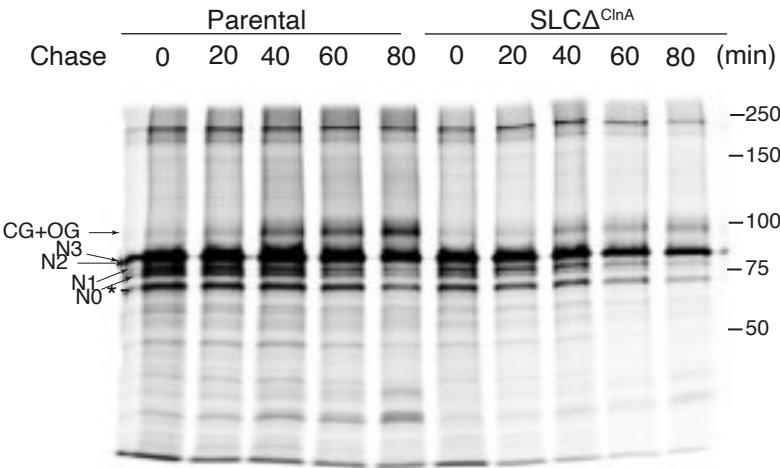

Supplement: Supplementary file 4 [file LSA-2026-03679_SdataF5.pdf]
