# Supplementary material for: Metabolite import by SLC33A1 is required for ATF6 activation during endoplasmic reticulum stress
Source: Life Sci Alliance. 2026 Apr 17;9(6):e202603679. doi: 10.26508/lsa.202603679 (PMC13090131; doi:10.26508/lsa.202603679)
Supplement: Supplementary file 5 [file LSA-2026-03679_TableS1.docx]

# Key Resources Tables

| **REAGENT or RESOURCE** | **SOURCE** | **IDENTIFIER** |
| --- | --- | --- |
| **Antibodies** | | |
| Monoclonal Mouse anti-FLAG M2 | Sigma | RRID:AB_262044 |
| **Chemicals, Peptides, and Recombinant Proteins** | | |
| EndoH | NEB | Cat# P0702S |
| PNGase | NEB | Cat# P0704S |
| O-Glycosidase amp Neuraminidase Bundle | NEB | Cat# E0540S |
| DMEM | Sigma | Cat#D6429 |
| Penicillin/Streptomycin | Sigma | Cat#P0781 |
| L-glutamine | Sigma | Cat#G7513 |
| Non-essential amino acids solution | Sigma | Cat#M7145 |
| HyClone II Serum | Thermo Fisher Scientific | Cat# SH30066.03 |
| Nutrient Mixture F12 | Sigma | Cat#N4888 |
| Lipofectamine LTX | Thermo Fisher Scientific | Cat#A12621 |
| ChromoTek GFP-Trap® Agarose | Thermo Fisher Scientific | Cat# Tag-20 |
| Tunicamycin | Melford | Cat# T2250 |
| 2-Deoxyglucose (2DG) | ACROS Organics | Cat# D6134 |
| Thapsigargin | Calbiochem | Cat# CAS 67526-95-8 |
| EDTA-free Protease inhibitor Cocktail | Roche | Cat#11873580001 |
| DMEM (-Glu/-Met/-Cys) | Gibco | Cat#21013024 |
| Easy TagTM Express ^35^S Protein Labelling Mix | Perkin-Elmer | NEG072007MC |
| 4μ8C | Tocris Bioscience | Cat#4479 |
| Anti-FLAG M2 Affinity Gel | Sigma | Cat#F3165 |
| MycoAlert (TM) Mycoplasma Detection Kit | Lonza | Cat# LT07-118 |
| TRIzolä Reagent | Ambion/Invitrogen | Cat#15596026 |
| PureLinkä RNA mini kit | Invitrogen | Cat#12183018A |
| RevertAid Reverse Transcriptase | Thermo Scientific | Cat# EP0441 |
| **Deposited Data** | | |
| Raw and analyzed data | Tung et al., 2024 | GEO: GSE254745 |
| **Experimental Models: Cell Lines** | | |
| Hamster: CHO-K1_XBP1s::mCherry & BiP::GFP (IRE1/ATF6α dual UPR reporter, parental) | Tung et al., 2024 | XC45-6S |
| Hamster: CHO-K1_XBP1s::mCherry & BiP::GFP *Slc33a1*∆^ClnA^ (KO) | This study | *Slc33a1*∆^ClnA^ |
| Hamster: CHO-K1_XBP1s::mCherry & BiP::GFP *Slc33a1*∆^ClnH^ (KO) | This study | *Slc33a1*∆^ClnH^ |
| Hamster: CHO-K1_XBP1s::mTurquoise & CHOP::GFP (IRE1/PERK dual UPR reporter, parental) | This study | S21 |
| Hamster: CHO-K1_XBP1s::mTurquoise & CHOP::GFP *Slc33a1*∆^ClnA.1^ (KO) | This study | *Slc33a1*∆^ClnA.1^ |
| Hamster: CHO-K1_XBP1s::mTurquoise & CHOP::GFP *Slc33a1*∆^ClnE^ (KO) | This study | *Slc33a1*∆^ClnE^ |
| Hamster: CHO-K1_XBP1s::mTurquoise & CHOP::GFP *Nat8∆*^ClnI^ (KO) | This study | *Nat8∆*^ClnI^ |
| Hamster: CHO-K1_XBP1s::mTurquoise & CHOP::GFP *Nat8b∆*^ClnB^ (KO) | This study | *Nat8b∆*^ClnB^ |
| Hamster: CHO-K1 3xFLAG-mGL_ATF6α knock-in cells | Tung et al., 2024 | 2K cells |
| Hamster: CHO-K1 3xFLAG-mGL_ATF6α knock-in cells  *Slc33a1*∆^ClnA9^ (KO) | This study | *Slc33a1*∆^ClnA9^ |
| Hamster: CHO-K1 GFP-cgATF6⍺LD_S1P&S2P^mut^ | Tung et al., 2024 | UK3122 cells |
| Hamster: CHO-K1 GFP-cgATF6⍺LD_S1P&S2P^mut^  *Slc33a1*∆^ClnK^ (KO) | This study | *Slc33a1*∆^ClnK^ |
| **Oligonucleotides** | | |
| *cgSlc33a1*_sgRNA1_ex1_1S *CACCGACAGTCACTCGGACTTGGT* | This study | 3072 |
| *cgSlc33a1*_sgRNA1_ex1_2AS *AAACACCAAGTCCGAGTGACTGTC* | This study | 3073 |
| *cgSlc33Aa1*_sgRNA2_ex1_1S *CACCGCGGTTTCAGCCTCAACCCA* | This study | 3074 |
| *cgSlc33a1*_sgRNA2_ex1_2AS *AAACTGGGTTGAGGCTGAAACCGC* | This study | 3075 |
| cg*Nat8b*_sgRNAg1_ex2_1S *CACCGGACTCTCCTGTCCTTATTTG* | This study | 3312 |
| cg*Nat8b*_sgRNAg1_ex2_1AS *AAACCAAATAAGGACAGGAGAGTC* | This study | 3313 |
| cg*Nat8b*_sgRNAg2_ex2_1S *CACCGGTCCTCCCAGCATCGAGGAC* | This study | 3314 |
| cg*Nat8b*_sgRNAg1_ex2_1AS *AAACGTCCTCGATGCTGGGAGGAC* | This study | 3315 |
| cg*Nat8*_sgRNAg1_ex1_1S *CACCGGTGTGTGTGGCTCAGGTTTC* | This study | 3316 |
| cg*Nat8*_sgRNAg1_ex1_1AS *AAACGTCCTCGATGCTGTGAGGAC* | This study | 3317 |
| cg*Nat8*_sgRNAg2_ex1_1S *CACCGGTCCTCACAGCATCGAGGAC* | This study | 3318 |
| cg*Nat8*_sgRNAg2_ex1_1AS *AAACGTCCTCGATGCTGTGAGGAC* | This study | 3319 |
| cg*Casd1* -sgRNA8_ex2_1 S  *CACCGGTTTCTTGAGCAATTAGTT* | This study | 3410 |
| cg*Casd1*-sgRNA8_ex2-1AS *AAACAACTAATTGCTCAAGAAACC* | This study | 3411 |
| cg*Xbp1*.19S5  *GGCCTTGTAATTGAGAACCAGGAG* | Neidhardt et al., 2023 | 1470 |
| cg*Xbp1*.14AS  *GAATGCCCAAAAGGATATCAGACTC* | Neidhardt et al., 2023 | 5 |
| cg*Rpl27*.1S  *AGGAAGTGAAGCCACCGCTCC* | Neidhardt et al., 2023 | 3248 |
| cg*Rpl27*.1AS  *AGGGCGGTCTGAAGTGCCAT* | Neidhardt et al., 2023 | 3249 |
| Mm*Hsp5a*_1S  *CCGAGGAGGAGGACAAGAAG* | This study | 28 |
| Mm*Hsp5a*_2AS  *CACATACGACGGCGTGATGC* | This study | 29 |
| **Recombinant DNA** | | |
| EGFP_cgATF6_343-659_N381F,P348L,LL408/9VV_Streptag_pCEFL_puro | This study | UK3116 |
| cgSLC33A1_g1_Ex1_pSpCas9(BB)-2A-mTurquoise | This study | UK2992 |
| cgSLC33A1_g2_Ex1_pSpCas9(BB)-2A-mTurquoise | This study | UK2993 |
| cgSLC33A1_g1_Ex1_pSpCas9(BB)-2A-mCherry | This study | UK3044 |
| cgSLC33A1_g2_Ex1_pSpCas9(BB)-2A-mCherry | This study | UK3045 |
| cgNAT8_sgRNA_g1_Ex1_pSpCas9(BB)-2A-mCherry | This study | UK3046 |
| cgNAT8_sgRNA_g2_Ex1_pSpCas9(BB)-2A-mCherry | This study | UK3047 |
| cgNAT8b_ex2_g1_pSpCas9(BB)-2A-mTurquoise | This study | UK3159 |
| cgNAT8b_ex2_g2_pSpCas9(BB)-2A-mTurquoise | This study | UK3160 |
| cgNAT8_sgRNA_g2_Ex2_pSpCas9(BB)-2A- mTurquoise | This study | UK3162 |
| **Software and Algorithms** | | |
| MAGeCK | Li et al., 2014 | PMID:25476604 |
| Metascape | Zhou et al., 2019 | PMCID:6447622 |
| FlowJo | BD | https://www.flowjo.com/ |
| Fiji (ImageJ 1.53c NIH) | Schindelin et al., 2012 | https://imagej.nih.gov/ij/ |
| Prism V8 | GraphPad | N/A |
| Volocity V6.3 | Perkin Elmer | N/A |
